# Time Required to Retreat Carrier-Based Obturation: Comparison Between Two Techniques at Two Levels of Experience

**DOI:** 10.3390/dj14030173

**Published:** 2026-03-17

**Authors:** Matteo Salvadori, Elisabetta Audino, Miriam Facchinetti, Vikas Kumar, Mario Alovisi, Luca Visconti, Stefano Salgarello

**Affiliations:** 1 Department of Medical and Surgery Specialties, Radiological Sciences and Public Health, School of Dentistry, Brescia University, 25123 Brescia, Italym.facchinetti011@unibs.it (M.F.); stefano.salgarello@unibs.it (S.S.); 2 Department of Surgical Sciences, Dental School, University of Turin, 10126 Turin, Italy; mario.alovisi@unito.it

**Keywords:** Thermafil retreatment, carrier-based obturation, carrier removal, braiding technique, Reciproc, 3D-printed teeth

## Abstract

**Background/Objectives:** This study aims to compare two techniques for the removal of Thermafil obturators, evaluating the influence of operator experience in two different typologies of samples. **Methods:** Sixty single-rooted extracted teeth with round canals and sixty 3D-printed teeth reproducing a maxillary central incisor were obturated with Thermafil obturators. Retreatment was undertaken under a dental operating microscope by an experienced endodontist and a novice operator using either the braiding technique or Reciproc. The removal time was recorded. **Results:** Considering natural teeth, seven failures were registered, and 60 carriers were removed successfully (90%). Removal time was significantly shorter for the experienced operator than for the novice (Braiding technique: *p* < 0.001; Reciproc: *p* = 0.001). No statistically significant difference emerged in the expert operator between braiding and reciprocating techniques (*p* = 0.403), while a longer carrier removal time emerged in the novice operator using the manual instrumentation (*p* = 0.019). Considering 3D-printed teeth, eight failures were registered, and 60 carriers were removed successfully (88%). There was no significant difference in removal time between novice and experienced operators. Carrier removal time was significantly lower in the braiding technique for the novice compared to the experienced operator (*p* = 0.017). This difference was not observed for the reciprocating instrumentation (*p* = 0.244). Regarding experience, in both operators, removal time was shorter with reciprocating instrumentation than with the braiding technique (*p* < 0.001). **Conclusions:** The braiding technique and Reciproc are effective in the retreatment of straight, round-section canals filled with Thermafil. Within the limits of this in vitro study, restoration of the working length can be undertaken quickly and with favourable outcomes. Experience significantly affects the removal time of carrier-based obturations. The removal technique did not influence retrieval time in the experienced operator, while the Reciproc proved to be an effective aid for the novice operator.

## 1. Introduction

Non-surgical endodontic retreatment of Thermafil obturation is a technically challenging procedure with limited predictability [1]. The primary difficulty involves the safe and complete removal of the plastic carrier, an attempt sometimes followed by complications [2,3,4,5,6,7,8,9]. Unsuccessful removal may occur and, in the presence of bacteria, may negatively affect the clinical outcome of endodontic treatment. For this reason, manual stainless-steel instruments (K-file, H-file) and mechanical Ni-Ti instruments (used with continuous rotation or reciprocation) have been investigated to determine their efficiency and strengths in retreating carrier-based obturation.

Manual instrumentation aims to isolate the plastic carrier from the surrounding gutta-percha to enable its removal, a favourable situation as it reduces debris production. This approach may be combined with the use of solvents [2,3,4,10], heat carriers [11], or Gates–Glidden drills [12], despite the possibility of unintentionally splitting the carrier. Conversely, mechanical retreatment using Ni-Ti instruments fragments the root canal filling without prior isolation of the carrier. This approach reduces retreatment time [5,6,7,8,13,14], allows for the effective management of curved or complex canal anatomies [5,6,7,8,9], although it may be associated with the risk of perforation, instrument fracture, or weakening of the canal walls. To overcome these limitations, additional techniques have been proposed. The Frag Remover, a wire-loop technique, could minimise root dentin removal, especially in the apical third [9]. A combination of microscope and ultrasonic tips may improve carrier engagement and anchorage, preventing the risk of separation [15]. However, these procedures can be challenging and time-consuming and may require advanced manual skills.

Despite the numerous methods suggested in the literature, many studies do not clarify which technique to adopt in relation to the clinical context and the operator’s experience. Furthermore, no hybrid technique combining the benefits of both manual instrumentation (complete removal of the carrier) and mechanical instrumentation (reduced operating times) was investigated. In relation to this lack of knowledge, the present study investigated two methods of carrier-based obturation retreatment (braiding technique vs. mechanical instrumentation with Reciproc) at two levels of expertise (expert vs. novice). The variable analysed was the retreatment time measured in successfully completed tests. Two substrates were considered: natural and 3D-printed teeth. The latter have become widely used in endodontics due to their easy availability and the standardisation criteria attributed to them [16,17,18,19]. 3D-printed teeth can faithfully reproduce numerous anatomical configurations and, compared to resin blocks, allow the dental crown to remain intact, improving the quality of the simulation. In the absence of studies evaluating the retreatment of carrier-based fillings in 3D replicas, the authors included them in this experimental work to evaluate their potential use in preclinical teaching. For each type of sample, the null hypotheses were as follows:

1. No statistically significant difference in removal time between the modified braiding technique and the reciprocating instrumentation, considering the individual operator.
2. No statistically significant difference in removal time between novice and expert operators, considering the techniques investigated. A null hypothesis was formulated between the two different types of samples.
3. No statistically significant difference in removal time between natural teeth and 3D-printed teeth, considering different removal techniques and expertise.

## 2. Materials and Methods

The experimental procedures were carried out by two operators with different levels of clinical experience. The expert operator (E) was an endodontist with experience in tutorship at the University of Brescia. The novice operator (B) was a sixth- and final-year dental student trained in the basic use of the microscope. Theoretical training on carrier-based filling retreatment and theoretical–practical training on gutta-percha retreatment with manual/rotary instruments were provided in the fifth year of the degree course.

Each operator was randomly assigned two experimental groups (n = 30) based on the type of substrate used: natural teeth (N) and 3D-printed teeth (P). Each group was further subdivided into two subgroups (n = 15) according to the retreatment technique employed: braiding technique with a Hedstroem-file (H) and mechanical instrumentation with Reciproc (R).

### 2.1. Sample Selection

Two different samples were selected:

1. Natural teeth
  Sixty single-rooted extracted teeth with intact roots and round canals were selected after radiographic examination, excluding those with curved or oval canals. Teeth were extracted for reasons not related to this study: informed consent and anonymisation were guaranteed by hospital procedure. The original canal diameter of each tooth was unknown. To simulate a clinical scenario, only teeth with a total length between 19 and 21 mm were included.
2. 3D-printed simulators
  Sixty 3D-printed teeth (REPLICA 1A; Brains, Gussago, Italy) reproducing a maxillary central incisor, characterised by an unopened access cavity and a straight canal, were used. The specimens were coronally sectioned to obtain a total length between 19 and 21 mm.

### 2.2. Canal Preparation and Obturation

Access cavity preparation, canal shaping, and obturation were performed by a single operator using 4.5× magnification loupes (Orascoptic, Madison, WI, USA), with the samples positioned on a Protrain (Dentsply Sirona, Tulsa, OK, USA).

A traditional access cavity was prepared using a round diamond bur (ZR801L012; Komet Dental, Lemgo, Germany) and an ultrasonic tip (CAP1; Satelec Acteon, Mérignac, France). After the determination of the working length, canals were shaped using the ProTaper Next system (Dentsply Maillefer, Ballaigues, Switzerland) up to an apical size of #30, taper 07, with copious irrigation using 5% sodium hypochlorite (Ogna, Muggiò, Italy). Apical patency was maintained using a #10 K-file.

Apical finishing, according to the step-back technique, was verified using manual NiTi instruments sizes #30, #35, and #40 (Nitiflex; Maillefer, Ballaigues, Switzerland). After drying, the canal walls were coated with AH Plus sealer (Dentsply DeTrey, Konstanz, Germany) using paper points.

Canal obturation was performed using Thermafil obturators size #30 with 0.04 taper (Dentsply Maillefer, Ballaigues, Switzerland) according to the manufacturer’s recommendations. The obturators were heated in a Thermaprep Plus oven (Dentsply Maillefer, Ballaigues, Switzerland) at 180 °C and introduced to the predetermined working length. After cooling the gutta-percha, the carrier was sectioned at the canal orifice using a round bur (K1SM205012; Komet, Lemgo, Germany) under dry conditions, leaving a remaining obturation length of 15 mm. Size and taper in the coronal canal portion were standardised at 1.20 mm, corresponding to the last working diameter of the Protaper Next instrument.

The access cavity was temporarily sealed with Cavit (3M ESPE, Seefeld, Germany). All samples were stored at 37 °C and 100% humidity for 30 days.

### 2.3. Retreatment Procedures

Following the removal of the temporary restoration, each operator was randomly assigned 30 natural teeth and 30 3D-printed teeth. The samples were positioned in a Protrain (Simit Dental, Mantova, Italy) and isolated with a rubber dam. ProTrain is a device designed specifically for endodontic training and allows the operator to perform endodontic therapy on extracted teeth. The extracted tooth or 3D-printed tooth is locked by a ring nut to allow the operator to perform all the operations necessary for a correct endodontic treatment. Non-surgical retreatment was performed in random order to reduce the effect of possible skills improvement on results. All the procedures were performed using a microscope (M320; Leica, Singapore).

The procedure was considered successful if the working length was re-established. If this outcome was not achieved, the test was classified as a failure and repeated using a new sample (natural tooth or 3D-printed tooth, according to the protocol described above. Failed samples were not considered in the statistical analysis.

### 2.4. Retreatment with Reciprocating Instrumentation

In the first subgroup, filling removal was attempted using a reciprocating R25 instrument (Reciproc; VDW, Munich, Germany) mounted on an X-Smart Plus motor (Dentsply Maillefer, Ballaigues, Switzerland). The operators used the Reciproc instrument with a pecking motion, ensuring penetration into the carrier by applying firm apical pressure, without irrigation. The flutes were cleaned of debris after every three pecking movements. Each instrument was replaced after five uses.

The procedure was continued until the re-establishment of the working length and confirmation of apical patency with a #10 K-file. The time required to reach the predetermined working length was recorded in seconds. Failed attempts and complications were documented.

### 2.5. Removal with Braiding Technique

In the second subgroup, the removal of the filling was attempted using #40 Hedstroem files (Dentsply Maillefer, Ballaigues, Switzerland), screwed around the carrier according to the braiding technique [10], following the application of 1 mL of solvent (Bio Orange, Muggiò, Italy).

To increase the effectiveness of Hedstroem files, the operators first removed the coronal gutta-percha surrounding the carrier using a heat-treated continuous-rotation instrument with a #30.07 tip (HyFlex Remover; Coltene/Whaledent AG, Altstätten, Switzerland) mounted on a Tri Auto ZX 2 endodontic motor (J. Morita Co., Kyoto, Japan) and operated at a speed of 1000 rpm. The rotating instrument tip was initially placed in contact with the coronal gutta-percha for 3 s to induce softening through frictional heat. Subsequently, using repeated pecking motions and applying firm apical pressure, the instrument was advanced to 6 mm below the canal orifice. The Hyflex instrument was replaced for each group or in case of fracture.

This procedure was repeated circumferentially around the carrier until sufficient space was created to allow insertion of two Hedstroem files, enabling their cutting blades to more effectively engage the carrier. The braiding technique was then performed by rotating the Hedstroem files three times around the carrier; their position and interlacing were verified under the operating microscope. Coronal traction was then applied in an attempt to remove both the manual instruments and the carrier as a single unit.

The procedure was repeated until complete removal of the carrier and the absence of carrier fracture was confirmed. If one or both files showed signs of deformation, they were immediately replaced; otherwise, the files were replaced after every three uses. Subsequently, a #10 K-file was used to reach the working length and confirm apical patency.

The procedure was continued until re-establishment of the working length and confirmation of apical patency with a #10 K-file. The time required to reach the predetermined working length was recorded in seconds. Failed attempts and complications were documented.

### 2.6. Statistical Analysis

Given the skewed distribution of working length restoration time assessed using the Shapiro–Wilk test, descriptive statistics are presented as median and interquartile range (IQR). Inferential analysis was performed using linear mixed-effects models to evaluate the effects of operator experience and retreatment technique. Experience and technique were included as fixed effects, and their interaction term was tested. Although the raw outcome showed deviation from normality, the distribution of model residuals was assessed graphically. Given the robustness of linear mixed-effects models to moderate deviations from normality, the parametric modelling approach was retained. Adjusted *p*-values were derived from model-based marginal contrasts, and post hoc comparisons were corrected using Bonferroni adjustment to control for multiple testing. Box-and-whisker plots were used to illustrate the median and interquartile range (IQR). Associations between operator experience or retreatment technique and complication incidence were assessed using Fisher’s exact test. Statistical significance was set at 5% (*p* < 0.05). All analyses were performed using STATA 18 (StataCorp, College Station, TX, USA).

## 3. Results

### 3.1. Group N: Natural Tooth

**Figure 1:**
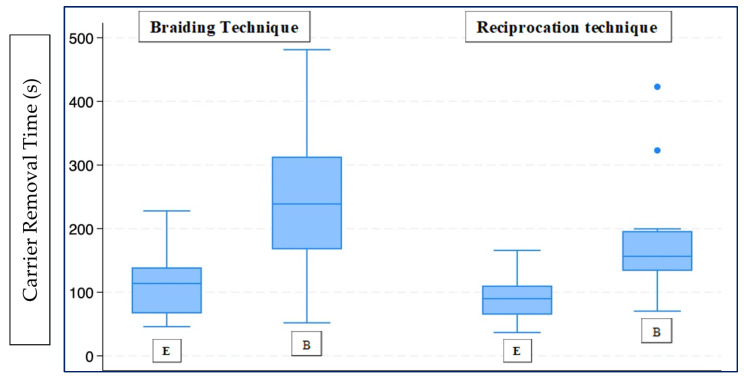
Box plot for Group N. E (experienced operator), B (novice operator).

Figure 1 summarises the removal time of the carrier with the braiding and Reciproc techniques in natural teeth. The null hypothesis, according to which there is no significant difference in removal time between novice and experienced operators, considering the techniques investigated, was rejected. In fact, the carrier removal time was significantly higher in the braiding technique for the novice (B = 238.75 s; 167.42–313.12) compared to the experienced operator (E = 113.74 s; 66.68–139.06) (*p* < 0.001). This difference was also observed for the reciprocating technique, which showed a longer removal time for the inexperienced operator (B = 156.55 s; 133.59–196.19) compared to the experienced operator (E = 89.86 s; 64.74–110.52) (*p* = 0.001). The null hypothesis that there is no significant difference in removal time between the braiding and reciprocating techniques was accepted for the experienced operator and rejected for the novice operator. Comparing the carrier removal times of the two techniques, no statistically significant difference emerged in the expert operator between Hedstroem and Reciprocating (*p* = 0.403), while a longer carrier removal time emerged in the inexperienced operator using the braiding technique (*p* < 0.05).

### 3.2. Group P: 3D-Printed Tooth

**Figure 2:**
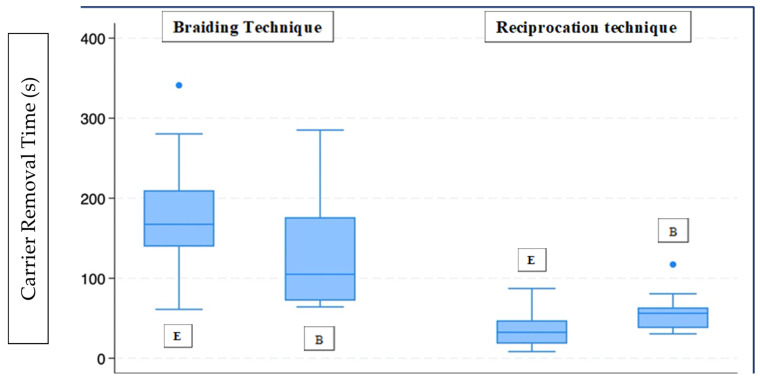
Box plot for Group P. E (experienced operator), B (novice operator).

Figure 2 summarises the removal time of the carrier with the braiding and Reciproc techniques in 3d-printed teeth. The null hypothesis that there is no significant difference in removal time between novice and experienced operators was rejected for the reciprocating technique and accepted for the braiding technique. Carrier removal time was significantly lower in the braiding technique for the inexperienced operator (B = 104.9 s; 72.04–176.23) compared to the experienced operator (E = 167.33 s; 139.55–209.86) (*p* = 0.017). This difference was not observed for the reciprocating instrumentation (*p* = 0.244). The null hypothesis that there is no significant difference in removal time between braiding and reciprocating techniques was accepted for both operators. Comparing the carrier removal times of the two techniques with respect to operator experience, a statistically significant difference emerged between the braiding and Reciprocating techniques in both the experienced operator (Braiding technique: 167.33 s, 139.55–209.86; Reciprocating: 32.4 s, 18.32–47.33, *p* < 0.001) and inexperienced operators (Braiding technique: 104.9 s, 72.04–176.23, Reciprocating: 56.17 s, 37.85–63.42, *p* < 0.001).

Table 1 summarises the complications in relation to the operator and the technique used, while Table 2 specifies the type of complication. Fifteen failed tests were recorded following complications that required replacement of the sample. A total of 67 natural teeth and 68 3D-printed teeth were used. Considering Group N, seven failures were recorded, and 60 carriers were successfully removed (90%); eight failures were recorded, and 60 carriers were successfully removed (88%) in Group P. Statistical analysis revealed no correlation between the removal outcome and the retreatment technique (*p* = 0.192); similarly, it did not show any correlation between the removal outcome and the operator’s experience, considering the single technique (Braiding technique, *p* = 0.165; Reciproc, *p* = 0.526). Of a total of 135 samples, 65 were attempts using the braiding technique and 70 using the Reciproc instruments. Considering the braiding technique, the only complication was carrier separation (5/65–7.7% of teeth). The reciprocating instrumentation recorded a greater number and variability of complications. Respectively, the complications included one instrument fracture (1/70–1.4%), one carrier separation (1/70–1.4%), one root perforation (1/70–1.4%), two apical transportations (2/70–2.9%), and five ledges (5/70–7.1%).

### 3.3. Group P vs. Group N

The null hypothesis, according to which there is no significant difference in removal time between natural teeth and 3D-printed teeth, considering different removal techniques and expertise, was rejected. Removal time was significantly longer in Group N than in Group P for the experienced (Reciprocation technique: *p* < 0,001; N = 89.86 s; 64.74–110.52; P = 32.4 s, 18.32–47.33) and the novice operator (Braiding technique: *p* < 0.05; N = 238.75 s; 167.42–313.12; P = 104.9 s; 72.04–176.23/Reciprocation technique: *p* = 0.001; N = 156.55 s; 133.59–196.19; P = 56.17 s, 37.85–63.42). Removal time was significantly longer in Group P for the experienced operator when limited to the braiding technique (*p* < 0.05; N = 113.74 s; 66.68–139.06; P = 167.33 s; 139.55–209.86).

## 4. Discussion

Disassembling is a delicate non-surgical retreatment procedure whose purpose is to empty the canal contents to ensure access of endodontic instruments and irrigants to the apical region. The nature and extent of endodontic materials, in relation to canal anatomy, determine the difficulty and influence the time required for the operative phases. In the case of the Thermafil obturator, the critical issue is the presence of the plastic support, which can prevent the working length from being restored and cause complications when attempting removal. [15]. Although many methods have been investigated, this in vitro study aims to clarify which technique to adopt in teeth with straight canal anatomy and in relation to the operator’s experience, comparing the braiding technique and Reciproc for the first time, on both natural (N) and 3D-printed teeth (P).

The teeth selected for this study were characterised by a straight canal, correctly shaped and filled, and an intact dental crown in order to faithfully reproduce a simple clinical scenario, ideal for preclinical training. Time to restore the working length was measured as root filling removal end-point: it is preferable for root canal filling removal to be performed quickly, as the less time taken, the more time can be devoted to chemical cleaning, with a potential improvement in the outcome [7,8,9,10,11,12,13,14,15,16,17,19]. Longer sessions can also lead to increased stress, both for the patient, with reduced compliance, and for the operator, with a potential risk of iatrogenic errors. Furthermore, from an economic perspective, longer treatment times can mean increased costs for the patient and the dentist [18].

The *braiding* and Reciproc techniques proved to be effective and reliable for removing carrier-based fillings from straight canals. Considering the total number of natural teeth and the anatomy investigated, the retreatment of the carrier-based filling was safe and predictable in 90% (60/67) of the tests performed; similar values (88%-60/68) were also found for the total number of 3D-printed teeth. Failed tests, not considered for evaluating removal time, were fifteen in total, and more numerous for Reciproc than the braiding technique (Table 2; R = 10; H = 5), but no statistically significant difference was found. For natural teeth, the most frequent incident was carrier separation (n = 3, one of which was then removed), followed by external apical transport (n = 2) and perforation (n = 1), which occurred during the use of reciprocating instruments. These complications were reported in some previous studies [2,3,5,7,8,9] in which the incidence varied from 5% [2,5,8] to 20% [5] in the tests considered. On the contrary, the replica recorded a higher value for ledge in the middle third of the canal (n = 5), which occurred with the reciprocating technique and was attributable to its lower hardness of resin compared to carrier separation (n = 3). Only one reciprocating instrument was fractured out of a total of 60 samples, demonstrating the reliability of R25, both on natural teeth and on the replica, when used every five uses. Similarly, Hülsmann et al. [9] also found a low percentage of fractures (6.6%) with R25.

Considering time removal in Group N, the type of instrumentation did not affect retreatment time when the operator was experienced (*p* = 0.403); conversely, in the novice operator, Reciproc allowed for more efficient removal of carrier-based obturation (*p* = 0.019). This highlighted a significant and apparently counterintuitive aspect. Mechanical Ni-Ti instruments may not reduce the duration of disassembling. This was the case with the experienced operator; on the contrary, it made the beginner’s performance more efficient. It is likely that the braiding technique requires a longer time to learn, typical of manual instrumentation: only effective contact between the instrument blades and the plastic surface, at an adequate depth, can ensure easy removal of the carrier. The retreatment time with the braiding technique was significantly longer (*p* < 0.001) for the novice operator (B = 238.75 s; 167.42–313.12) than for the experienced operator (E = 113.74 s; 66.68–139.06). Similarly, the difference was significant (*p* = 0.001) for the reciprocating technique: the novice operator took longer (B = 156.55 s; 133.59–196.19) than the expert (E = 89.86 s; 64.74–110.52). The expert operator was twice as fast with manual instruments and almost twice as fast with reciprocating instruments. These results are intuitive and suggest that root canal retreatment is a technical procedure that requires tactile skills, which are developed over time through preclinical and clinical practice.

Although natural teeth represent the gold standard substrate for simulating clinical scenarios, their scarcity, the risk of cross-contamination, and the difficulty in standardising anatomy are factors that have favoured the spread of 3D-printed teeth, despite their higher cost [19]. They are widely used in both dental education and preclinical research, as 3D printers are now capable of quickly producing a large number of prototypes with identical anatomical characteristics [20]. For these reasons, this study investigated the retreatment of carrier-based fillings in relation to 3D-printed teeth, although these differed from natural teeth in terms of their lower hardness.

Considering time removal in Group P, the type of instrumentation affected retreatment time in both operators (*p* < 0.001): Reciproc recorded faster times compared to the braiding technique. The physical properties of the resin could allow Ni-Ti mechanical instruments to cut more efficiently than purely manual or hybrid instrumentation. The same mechanism could explain why shorter retreatment times occurred in group P compared to N, except for the experienced operator when performing the braiding technique. Ni-TI reciprocating instruments could be more effective in 3D-printed replicas, due to the lower hardness of the resin compared to dentin. This physical property is the main limitation of 3D-printed teeth [19,20], which has yet to be fully effective as a replica. Awaiting the development of more accurate replicas containing hydroxyapatite or ceramic particles [21,22], the data obtained must be transferred with caution to the clinical setting, possibly, as in this study, by comparing them with extracted teeth. Despite differences compared to natural teeth, the results of this study welcomed the use of 3D replicas, limited to straight canals, to compare the Thermafil retreatment methods or practice preclinical training. The replicas proved to be adequate for the experiment conducted. However, a very similar pilot study conducted by the same authors to investigate the retreatment of the carrier-based system showed that reciprocating instruments were not suitable for 3D replicas with curved canals due to the high incidence of ledges and perforations during testing, caused by the impact of the instrument blades against the softer walls in the curved areas.

**Figure 3:**
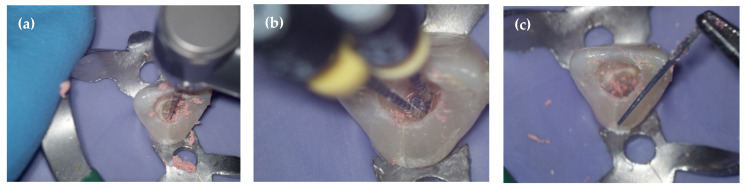
(**a**) Removing gutta-percha with Hyflex Remover #30.07 facilitated the positioning of H-file #40. (**b**) Two Hedstroem files #40 are placed around the carrier. (**c**) The braiding technique allows the plastic carrier to be removed entirely.

Although it has been rarely studied in the literature, the braiding technique is an effective and recommended method for the complete removal of plastic carriers, as observed in this study [Figure 3]. Hedstroem files were highly efficient and still a valuable aid in the retreatment of carrier-based obturation. They have enabled to achieve shorter times than in similar studies, previously conducted with manual k-file instruments [3,4,11]. There may be two reasons for this: the greater cutting capacity of H-files [23] and the use of continuous rotation mechanical instruments in the initial stages of retreatment. Indeed, the data collected suggest that the combination of manual and mechanical instruments allows for the efficient selective removal of the carrier, upon which the success of retreatment may depend [24].

The Hyflex Remover system consists of a single instrument with a triangular section made of martensitic C-Wire alloy. It has good resistance to torsional fatigue, which is superior to the austenitic Ni-Ti used in retreatment (M-Two and ProTaper Retreatment Files) [25]. The use of Hyflex Remover in this study, in combination with manual instruments, may have contributed to reducing retreatment time by optimising the removal of the coronal third gutta-percha. The same instrument has also demonstrated its effectiveness in Thermafil retreatment in other ex vivo studies, where it was used in continuous rotation up to 3 mm from the WL [26,27]. In this study, Hyflex Remover demonstrates good resistance and effectiveness, suggesting its use as an aid to manual instrumentation. More specifically, the authors pointed out that, in the braiding technique, the use of a microscope and the choice of a martensitic retreatment instrument with reduced primary cutting capacity prevented carrier laceration: in the only case where this incident occurred (1/60), the remaining portion was still removed.

Great care must be taken to selectively remove the gutta-percha adjacent to the carrier without weakening the latter, exposing it to the risk of separation. In this regard, Ruíz-Piñón et al. [15], in a recent case report, presented a new technique for removing the carrier in order to reduce the risk of fracture. Under a microscope, an ultrasonic insert with an active tip consumed the central part of the carrier, allowing the H-file instrument to penetrate the plastic. The operator used mosquito forceps to grasp the Hedstroem instrument and, applying force parallel to the tooth axis, attempted to remove the carrier. The technique described does not involve the removal of gutta-percha during the carrier removal phases; however, to achieve this goal, Piñón et al. recommended adequate magnification and an active ultrasonic tip, as well as noted a steep learning curve.

**Figure 4:**
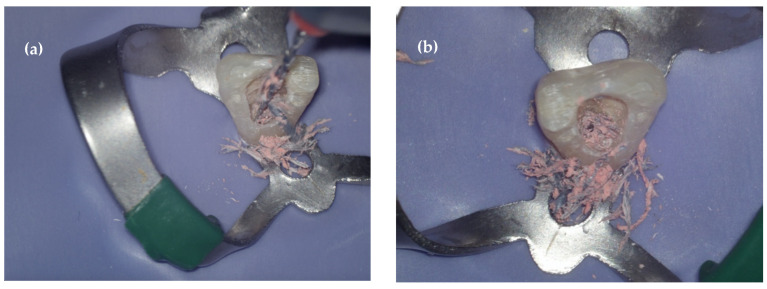
(**a**) Thermafil removal with Reciproc #25; (**b**) reciprocating instruments fragmented the plastic carrier across all samples analysed.

In addition to removing the plastic carrier entirely, another valid operating procedure is to break it up using a reciprocating instrument. Reciproc has fragmented the plastic carrier across all samples analysed [Figure 4], allowing the working length to be restored. Reciproc is an instrument in M-Wire alloy, with a non-active tip and an ‘S italica’ section, effective in removing carrier-based obturation [9,21,28]. In this study, reciprocation recorded a longer average retreatment time than that reported in previous studies, which used continuous rotation with Pro Taper Retreatment in curved or moderately curved canals [6,7,8]. It is likely that the higher rotation speed used in previous studies (500 rpm) favoured the faster removal of Thermafil. This result was also found by Royzenblat & Goodell [5], who demonstrated the greater efficiency of ProFile 04 at higher speeds (1500 rpm > 300 rpm). Nevertheless, the results of the present study highlight not only the effectiveness but also the adequate resistance of the Reciproc instrument, which was used successfully in straight canals until the end of five tests, despite being presented as a single-use instrument.

The results of this research encourage the use of both techniques in the retreatment of carrier-based fillings. Round-section root canals, such as those of the upper incisors and canines, or the distobuccal and palatal canals of the maxillary molars, could be treated with ease, especially in straight trajectories. In light of the data collected, the authors recommend removing the plastic carrier using a hybrid technique rather than manual instruments alone, as this is a simple and quick method. On the other hand, Reciproc has been particularly efficient, especially for less experienced operators. Given its fracture resistance, it could be used safely in the retreatment of a multi-rooted element filled using the Thermafil technique.

The present study has some limitations. The canals, previously shaped with ProTaper Next 30.07, were filled with Thermafil 30.04. This may have facilitated the removal of the carrier, given the greater thickness of gutta-percha between the plastic and dentin. Secondly, the solvent was only used with the braiding technique, since retreatment with Reciproc is documented as dry [9]. By combining the solvent with the reciprocating technique, it is possible to further reduce operating times, but debris removal could be more difficult. An additional limitation of the study was the sample housing: the teeth were placed in Protrain and not in a phantom head (real scenario) as suggested by Ajina et al. [29] and Hülsmann [21]. Nevertheless, the use of a microscope by both operators ruled out the possibility of changing the working distance, maintaining the one normally used. Regarding the presence of gutta-percha on the walls, this study did not analyse this element, since Rodig et al. [8] highlighted that the parameter was not influenced by the retreatment technique. Further studies could investigate the retreatment methods illustrated in curved canals, comparing different apical shaping, both in natural teeth and in replicas whose hardness is acceptable for the tests to be carried out. Different preparation diameters, both in the coronal and apical third, could be compared to assess the difficulty of re-treating the Thermafil obturator.

## 5. Conclusions

The operator’s experience influences the retreatment time of carrier-based obturation, favouring the experienced clinician. In teeth with a straight canal anatomy, disassembling was safe and simple for both techniques analysed (braiding technique vs. reciprocating instrumentation). Reciprocating instrumentation served as an efficient aid for novice operators in the retreatment of Thermafil, especially when considering the average retreatment times and the relatively low and insignificant number of incidents. Economic considerations and subjective factors could influence the experienced clinician in the technique to be adopted, since the removal time was not affected by the method used (*p* = 0.403).

## Figures and Tables

**Table 1:** Incidence of complications.

	Total	Novice	Experienced	*p*-Value
**Braiding Technique**	5	4	1	0.165
**Reciproc**	10	6	4	0.526
***p*** **-value**	0.192	0.526	0.165	

**Table 2:** Type of complications.

	Braiding Technique	Reciproc
**Instruments fracture**	/	1
**Carrier separation**	5	1
**Apical transportation**	/	2
**Ledge**	/	5
**Radicular perforation**	/	1

## Data Availability

The data presented in this study are available on request from the corresponding author due to privacy.
